# Application of a New Dehydroascorbic Acid Reducing Agent in the Analysis of Vitamin C Content in Food

**DOI:** 10.3390/molecules26206263

**Published:** 2021-10-16

**Authors:** Artur Mazurek, Marzena Włodarczyk-Stasiak

**Affiliations:** Department of Analysis and Evaluation of Food Quality, Faculty of Food Science and Biotechnology, University of Life Sciences in Lublin, Skromna Street 8, 20-704 Lublin, Poland; marzena.stasiak@up.lublin.pl

**Keywords:** vitamin C, ascorbic acid, dehydroascorbic acid, tris(hydroxypropyl)phosphine, unithiol, tris(2-carboxyethyl)phosphine, validation

## Abstract

The analysis of total vitamin C content in food is most frequently performed by reducing dehydroascorbic acid to ascorbic acid, which is then assayed with the technique of high-performance liquid chromatography combined with spectrophotometric detection. Tris(2-carboxyethyl)phosphine is currently the only agent in use that efficiently reduces dehydroascorbic acid at pH < 2. Therefore, there is a continued need to search for new reducing agents that will display a high reactivity and stability in acidic solutions. The objective of the study was to verify the applicability of unithiol and tris(hydroxypropyl)phosphine for a reducing dehydroascorbic acid in an extraction medium with pH < 2. The conducted validation of the newly developed method of determining the total content of vitamin C using tris(hydroxypropyl)phosphine indicates its applicability for food analysis. The method allows obtaining equivalent results compared to the method based on the use of tris(2-carboxyethyl)phosphine. The low efficiency of dehydroascorbic acid reduction with the use of unithiol does not allow its application as a new reducing agent in vitamin C analysis.

## 1. Introduction

An optimal method of vitamin C determination should be based on the measurement of its total content (T_C_), being the sum of the contents of ascorbic acid (AA) and dehydroascorbic acid (DHAA) [1]. Simultaneous and direct detection of L-ascorbic acid and dehydroascorbic acid is a complex analytical problem, as those acids display strongly differing absorption, fluorescence, and electrochemical properties. In general, it is necessary to transform the oxygenated form of the acid to the reduced form, or vice versa, so that they are in the same form, as then it is possible to use a single detection technique. Concerning these issues, various analytical methods are used to determine the total content of vitamin C [2]. The determination of the total content of vitamin C through a reduction in dehydroascorbic acid to L-ascorbic acid, combined in the next step of analysis with the detection of the reduced form, is the most frequent method of performing the analysis with the use of HPLC. It is also possible to simultaneously determine the total content of vitamin C and dehydroascorbic acid using the so-called differential method [3]. In this case, the first step of the procedure is the analysis of L-ascorbic acid contained in the sample. Next, the quantitative reduction in dehydroascorbic acid is performed, followed by the determination of the total vitamin C content. The content of dehydroascorbic acid is determined by subtracting the initial content of L-ascorbic acid from the total vitamin C content. This type of procedure of analysis of dehydroascorbic acid is characterized by better selectivity and sensitivity than those using spectrophotometric detection in the low UV range [4].

The reduction reaction can be conducted at the sample preparation stage or after the chromatographic separation (post-column reduction) and before the detection. Dehydroascorbic acid is reduced with the use of reducing agents containing the thiol group: dimercaptopropanol [5,6], mercaptoethanol [7,8], cysteine [9], homocysteine [10,11], and recently the most frequently used dithiothreitol [12,13,14,15,16]. These reagents are stable and active only in neutral or slightly acidic pH. Vitamin C extraction from a sample is performed in solutions with low pH to ensure its stability, which causes the necessity of raising the pH of the extract before the reduction procedure. This stage complicates the preparation of samples for the analysis and can generate errors, as vitamin C undergoes accelerated degradation in neutral pH. This method was applied in vitamin C analysis according to the European standard EN 14130:2003. However, in 2010, the technical committee CEN/TC 275 Food analysis—Horizontal methods made the decision to withdraw the method without any substitution due to the obtainment of unreliable and non-credible results. Reagents reducing dehydroascorbic acid in an extraction medium with pH < 2 are sulfide ions and tris(2-carboxyethyl)phosphine (TCEP). Sodium hydrosulfide was used to determine the total content of vitamin C in citrus fruit juices [17]. 

Tris(2-carboxyethyl)phosphine is currently the only reagent in use that provides an efficient reduction in dehydroascorbic acid at the pH of a metaphosphoric acid solution [18,19,20,21,22]. Hence, the continuing need to search for new reducing agents that will display a high reactivity and stability in acidic solutions. The basis for undertaking a study on the application of tris-hydroxypropyl-phosphine (THP) as an agent reducing dehydroascorbic acid is that, like TCEP (and most DHAA reducing agents), it is used in research on proteins for the purpose of reducing disulfide bonds. In addition, tris-hydroxypropyl-phosphine displays a higher reactivity in relation to small molecules containing disulfide bonds compared to TCEP [23]. Another substance selected as a potential DHAA reducing agent is sodium salt of 2,3-Dimercapto-1-propanesulfonic acid (unithiol).

Unithiol is a chemical compound with metal-chelating properties. It is used as an antidote administered orally or intravenously in metal poisoning [24]. If unithiol could efficiently reduce dehydroascorbic acid, it would be a better reducing agent than TCEP, as it would additionally chelate metals, thus enhancing the stability of ascorbic acid. Research on the application of unithiol for reducing dehydroascorbic acid in the pH range of 1.25–1.3 was conducted as early as 1974. It was demonstrated that with a dehydroascorbic-acid-to-unithiol ratio of 1:500, at room temperature and the pH within the range of 1.25–1.3, the dehydroascorbic acid undergoes a complete reduction in 15 min. The results of that reaction were used as the basis for developing a method for determining DHAA applied in a study on rat liver and kidneys [25]. In another study, the reducing properties of unithiol were confirmed at concentrations of 2.4–71 mM in DHAA solution with pH 5.6. The duration of the reaction was 1.5 h [26]. Since the publication of those studies, there have been no new reports that would describe the use of unithiol in vitamin C analysis. 

Therefore, the objective of the study was to confirm the applicability of unithiol and tris-hydroxypropyl-phosphine for a reduction in dehydroascorbic acid in an extraction medium with pH < 2. Validation of the chromatographic method of vitamin C determination in food, based on a new DHAA reducing agent, was performed. A solution of metaphosphoric acid, most frequently used to extract vitamin C from samples of food products [3], was selected for the study.

## 2. Results and Discussion

### 2.1. Reduction Properties

**Figure 1:**
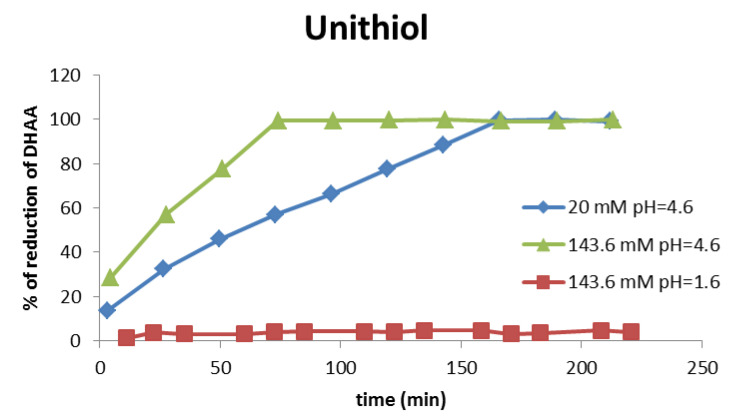
Degree of reduction in dehydroascorbic acid versus time, with the use of unithiol.

The results of the analysis of the reducing properties of unithiol with the concentration of 143.6 mM in metaphosphoric acid with pH 1.6 are presented in Figure 1. It was demonstrated that unithiol is not applicable for a reduction in dehydroascorbic acid at pH 1.6. In that environment, the level of reduced DHAA did not exceed 7%. The results obtained do not conform to the studies presented in the literature, even though the same ratio of DHAA to unithiol was used—1:500 [25]. Kozlov and L’vova [26] demonstrated that unithiol efficiently reduced dehydroascorbic acid in an environment with pH 5.6. Depending on the applied concentration of unithiol (from 2.4 to 71 mM), after 90 min of the reaction, the degree of reduction in DHAA varied from 91.2% to 94%. Figure 1 presents the results of the analysis of the reduction properties of unithiol with concentrations of 20 and 143.6 mM in the environment of an acetate buffer with pH 4.6. The time after which a quantitative reduction in DHAA was obtained at these concentrations was 165 and 74 min, respectively. These results support the earlier studies, but despite the efficient reduction in DHAA by unithiol at pH 4.6, the low efficiency of reduction at pH 1.6 does not allow its application as a new reducing agent in vitamin C analysis.

**Figure 2:**
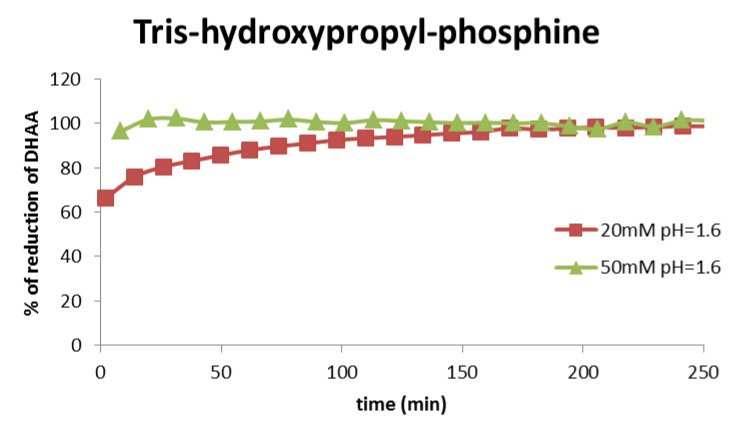
Degree of reduction in dehydroascorbic acid versus time, with the use of tris-hydroxypropyl-phosphine.

Figure 2 presents the results of the analysis of the reduction properties of tris-hydroxypropyl-phosphine with concentrations of 20 and 50 mM in the environment of metaphosphoric acid with pH 1.6. It was demonstrated that THP is an efficient dehydroascorbic acid-reducing agent; however, the time required for a complete reduction (above 200 min) in the case of application at the concentration of 20 mM is too long. Tris-hydroxypropyl-phosphine with a concentration of 50 mM reduces DHAA quantitatively as early as after 20 min. At that concentration, it can be used to develop a new procedure of vitamin C determination. In the case of analysis of actual samples of food products, the time of reduction was increased to 30 min. 

### 2.2. Method Validation

The selectivity, linearity and lower limit of detection and quantification of the analytical method are related to the applied chromatographic technique of measurement of ascorbic acid content. Therefore, the values of these parameters are identical to the validation parameters previously determined by us for the chromatographic method of ascorbic acid determination [21]. That method makes use of the best-so-far dehydroascorbic acid-reducing agent, tris(2-carboxyethyl)phosphine, and it is used in this study as the reference method. The modification of the method, consisting of the use of tris-hydroxypropyl-phosphine for the reduction in dehydroascorbic acid at the stage of sample preparation, does not affect the abovementioned validation parameters. For this reason, only the precision and accuracy of the newly developed analytical method were determined. 

#### 2.2.1. Precision

Table 1 presents the results of the analysis of food samples conducted with the chromatographic method using tris-hydroxypropyl-phosphine. In the case of analysis of ascorbic acid and the total content of vitamin C, the coefficient of variation varies in the range from 1.57% to 6.51%, and in the case of dehydroascorbic acid in the range from 11.58% to 89.14%. The values of the Horrat parameter of the analysis of ascorbic acid and total vitamin C content fall within the range conforming to the recommendations of AOAC [27] for interlaboratory studies and vary from 0.32 to 0.92. The newly developed method allows for obtaining the precise results of the assay of ascorbic acid content and the total content of vitamin C. In the case of dehydroascorbic acid determination, the values of the Horrat parameter fall within the range from 2.05 to 12.51, which shows that the analysis of that acid using the differential method is imprecise. It was demonstrated that DHAA assay with the differential method leads to the obtainment of imprecise results, which follows from the assumptions of that method and the law of propagation of uncertainty [21]. Table 1 also presents the results of analyses of food samples with the reference method using tris(2-carboxyethyl)phosphine. The precision of determination of the two methods was compared with the use of the Snedecor F-test. In the case of assays of the content of ascorbic acid and total content of vitamin C for all samples, the analysis of the results of the Snedecor F-test indicates an absence of statistically significant differences of standard deviations. Therefore, the method using tris-hydroxypropyl-phosphine is characterized by the same precision as the reference method. In the case of analysis of the content of dehydroascorbic acid, the Snedecor F-test indicates the occurrence of statistically significant differences only for samples of cauliflower, kissel and infant milk powder (Table 1) resulting from high values of the standard deviation of DHAA measurements related to the law of propagation of uncertainty [21].

#### 2.2.2. Accuracy

The accuracy of the obtained results was tested by analyzing a sample of a certified reference material, BCR 431, and comparing the obtained mean value with the certificated value. Table 1 presents the obtained data, which indicate an absence of statistically significant differences, both in the case of the new method using tris-hydroxypropyl-phosphine and the reference method. Table 2 presents a comparison of the results of analyses of ascorbic acid, the total content of vitamin C and dehydroascorbic acid acquired with the new method using tris-hydroxypropyl-phosphine, with the results obtained with the reference method. The results obtained do not display any statistically significant differences, indicating the equivalence of results obtained with both methods. However, imprecise analysis of dehydroascorbic acid suggests an absence of statistically significant differences for results that otherwise differ significantly from one another, e.g., 79.87 mg/100 g and 100.38 mg/100 g in the case of the sample of the certified reference material.

## 3. Materials and Methods

### 3.1. Materials

The test materials used in the study included juices (multi-vegetable, grapefruit, orange), fruits and vegetables (banana, kiwi, lemon, cauliflower, broccoli, cucumber, tomato, parsley tops), multi-vitamin syrup, instant kissel (powder) and infant milk powder, purchased from local retail shops. The certified reference material BCR-431 was purchased from the Institute of Reference Materials and Measurements (IRMM), European Commission Joint Research Centre.

### 3.2. Chemicals

**Figure 3:**
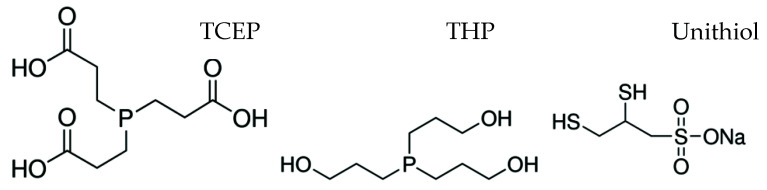
Chemical structure of tris(2-carboxyethyl)phosphine (TCEP), tris-hydroxypropyl-phosphine (THP) and unithiol.

L-ascorbic acid, dehydroascorbic acid and tris(2-carboxyethyl)phosphine (Figure 3) were purchased from the company Sigma-Aldrich (Saint Louis, MO, USA); tris-hydroxypropyl-phosphine (Figure 3) and metaphosphoric acid from Merck (Darmstadt, Germany); sodium salt of 2,3-dimercapto-1-propanesulfonic acid (unithiol, Figure 3) from Alfa Aesar (Kandel, Germany); and orthophosphoric acid from POCH S.A (Gliwice, Poland). All reagents complied with the quality standards required for analytical grade chemicals.

### 3.3. Procedure of Testing the Reduction Properties

The testing of the reducing properties of unithiol and tris-hydroxypropyl-phosphine was conducted through a reduction in dehydroascorbic acid with a concentration of 0.57 mM in an acetate buffer with pH 4.6 and in a 2% solution of metaphosphoric acid with pH 1.6. The applied concentration of unithiol was 20 mM and 143.6 mM, while that of tris-hydroxypropyl-phosphine was 20 and 50 mM. The progress of the reduction was monitored using chromatographic determination of the concentration of ascorbic acid. Due to the possibility of a decrease of DHAA concentration in the initial solution resulting from its uncontrolled degradation, the percentage reduction in DHAA was determined in relation to the result obtained from the reduction with tris(2-carboxyethyl)phosphine with a concentration of 20 mM.

### 3.4. Sample Preparation for Analysis

Sample preparation for analysis was performed in conformance with the method described in the paper by Mazurek and Jamroz [2]. In the case of DHAA reduction with the use of tris-hydroxypropyl-phosphine, the applied concentration was 50 mM. During all stages of the analyses, the temperature in the laboratory was maintained at below 25 °C. All analyses were performed in six replicates, and the results were expressed in mg of analyte per 100 g of fresh matter in the case of fruits and vegetables and in the other samples—per 100 g of the product.

### 3.5. HPLC Analysis

The analysis of L-ascorbic acid was performed using the reversed-phase high-performance liquid chromatography in conformance with the method described by Mazurek and Jamroz [21]. A diode-array detector was used for the detection of analytes. The separation was conducted in the conditions of isocratic elution. In the first step, the content of L-ascorbic acid in the sample was assayed, followed by the quantitative reduction in dehydroascorbic acid using a reducing agent. Then, the total content of vitamin C was determined. The content of dehydroascorbic acid was calculated by subtracting the initial content of L-ascorbic acid from the total content of vitamin C. The concentration of L-ascorbic acid in the extract was determined from the equation of the calibration curve plotted based on the results of the analysis of standard solutions. The identification of L-ascorbic acid was performed based on the time of retention and the UV spectrum of the reference substance. The analyses were performed with the use of a Varian (Palo Alto, CA, USA) HPLC system equipped with a diode-array detector (DAD, type 335), an isocratic pump (type 210), a dosing valve 7725i (Rheodyne, Cotati, CA, USA), a column thermostat, and a chromatographic column, Gemini 150 × 4.6 mm (3 μm C18) connected with a pre-column Gemini C18 4 × 3 mm (Phenomenex, Torrance, USA). The injection volume was 20 μL. The mobile phase was a solution of orthophosphoric acid at pH 2.8 pumped at a 0.6 mL/min flow. Chromatograms were recorded at 244 nm and a temperature of 30 °C.

### 3.6. Validation

The determination of the precision, comparison of the precision of the methods, the accuracy of the new method through the analysis of a sample of certificated reference material and the comparison of the results obtained with the use of the chromatographic reference method were performed in conformance with the method described by Mazurek et al. [28]. 

## 4. Conclusions

Contrary to earlier reports [25], unithiol is characterized by low reduction efficiency in relation to dehydroascorbic acid in environments with pH < 2. The new efficient DHAA reducing agent in such an environment is tris-hydroxypropyl-phosphine. The conducted validation of the newly developed method of determining the total content of vitamin C using tris-hydroxypropyl-phosphine indicates the suitability of the method for food analysis. In the case of the dehydroascorbic acid content analysis, imprecise results are obtained, resulting from the application of the differential method [21]. The results of analyses performed using the developed HPLC-THP method are characterized by a high level of agreement with the results obtained with the chromatographic reference method using tris(2-carboxyethyl)phosphine and suggest equivalence of the two methods. 

## Figures and Tables

**Table 1:** Comparison of ascorbic acid (AA), total vitamin C (T_C_) and dehydroascorbic acid (DHAA) values obtained by chromatographic method with the use of tris(2-carboxyethyl)phosphine (HPLC-TCEP) and tris(hydroxypropyl)phosphine (HPLC-THP) and statistical characteristics of the precision of determinations (F_cr_ = 5.05 for α = 0.05 and f = 5).

		HPLC-TCEP				HPLC-THP							
Sample	Analyte	Mean (mg/100 g)	s * (mg/100 g)	CV	H *	Mean (mg/100 g)	s * (mg/100 g)	CV	H *	F *	P *	U *	Accuracy
Multivegetable juice	AA	35.75	1.85	5.19	0.79	34.00	1.63	4.78	0.72	1.30	0.95	0.14	yes
	T_C_	38.07	1.59	4.18	0.64	36.64	0.78	2.13	0.32	4.14	0.96	0.09	yes
	DHAA	2.31	0.87	37.78	3.79	2.64	1.42	53.70	5.49	2.62	1.14	1.34	yes
Orange juice	AA	17.80	0.56	3.12	0.43	19.48	0.73	3.77	0.52	1.75	1.09	0.10	yes
	T_C_	23.43	0.85	3.62	0.51	25.06	0.72	2.87	0.41	1.39	1.07	0.09	yes
	DHAA	5.62	1.28	22.71	2.60	5.58	1.20	21.44	2.45	1.14	0.99	0.62	yes
Grapefruit juice	AA	28.08	0.92	3.28	0.48	28.48	0.63	2.20	0.32	2.16	1.01	0.08	yes
	T_C_	31.97	0.84	2.63	0.39	31.81	1.23	3.88	0.58	2.16	1.00	0.09	yes
	DHAA	3.89	1.34	34.54	3.75	3.33	1.31	39.46	4.18	1.04	0.86	1.04	yes
Parsley tops	AA	209.54	9.14	4.36	0.86	214.91	10.00	4.65	0.92	1.20	1.03	0.13	yes
	T_C_	226.03	10.88	4.81	0.96	235.44	6.87	2.92	0.59	2.50	1.04	0.11	yes
	DHAA	16.50	4.90	29.73	4.01	20.53	10.24	49.87	6.95	4.36	1.24	1.23	yes
Tomato	AA	13.73	0.99	7.18	0.94	14.24	0.51	3.61	0.48	3.67	1.04	0.16	yes
	T_C_	16.35	0.70	4.29	0.58	16.69	1.09	6.51	0.88	2.39	1.02	0.16	yes
	DHAA	2.61	1.41	53.97	5.51	2.45	1.14	46.48	4.70	1.53	0.94	1.43	yes
Broccoli	AA	97.18	2.93	3.01	0.53	103.41	4.33	4.19	0.74	2.19	1.06	0.10	yes
	T_C_	115.36	2.44	2.12	0.38	115.02	3.73	3.24	0.58	2.33	1.00	0.08	yes
	DHAA	18.18	4.20	23.08	3.16	11.61	5.42	46.73	5.97	1.67	0.64	0.92	yes
Cauliflower	AA	74.71	1.99	2.66	0.45	79.86	2.21	2.77	0.47	1.24	1.07	0.08	yes
	T_C_	98.30	2.52	2.56	0.45	103.71	4.10	3.95	0.70	2.65	1.06	0.10	yes
	DHAA	23.58	2.38	10.08	1.43	23.85	6.04	25.33	3.61	6.45	1.01	0.55	yes
Banana	AA	12.93	0.77	5.97	0.78	14.10	0.54	3.83	0.50	2.04	1.09	0.14	yes
	T_C_	16.71	0.63	3.79	0.51	17.40	0.56	3.24	0.44	1.27	1.04	0.10	yes
	DHAA	3.79	1.11	29.30	3.16	3.30	0.69	20.97	2.22	2.57	0.87	0.74	yes
Lemon	AA	86.87	2.96	3.41	0.59	88.00	2.91	3.31	0.57	1.03	1.01	0.10	yes
	T_C_	90.59	3.47	3.83	0.67	91.45	3.62	3.96	0.69	1.09	1.01	0.11	yes
	DHAA	3.72	2.15	57.75	6.22	3.45	3.08	89.14	9.49	2.05	0.93	2.09	yes
Cucumber	AA	6.16	0.41	6.62	0.77	6.51	0.33	5.06	0.59	1.53	1.06	0.17	yes
	T_C_	7.13	0.44	6.13	0.73	7.35	0.22	2.93	0.35	4.11	1.03	0.13	yes
	DHAA	0.98	0.33	33.90	2.99	0.84	0.33	39.05	3.36	1.01	0.86	1.03	yes
Instant kissel	AA	90.55	1.91	2.11	0.37	90.89	1.95	2.14	0.37	1.04	1.00	0.06	yes
	T_C_	92.92	2.51	2.70	0.47	93.78	2.41	2.57	0.45	1.09	1.01	0.07	yes
	DHAA	2.36	1.44	60.86	6.12	2.89	0.63	21.77	2.26	5.22	1.22	1.19	yes
Multivitamin syrup	AA	974.10	20.99	2.15	0.54	975.20	24.82	2.55	0.63	1.40	1.00	0.07	yes
	T_C_	1023.39	18.21	1.78	0.45	1021.33	22.79	2.23	0.56	1.57	1.00	0.06	yes
	DHAA	49.29	31.07	63.03	10.0	46.12	36.68	79.52	12.5	1.39	0.94	2.02	yes
Infant milk powder	AA	87.44	3.29	3.76	0.65	90.67	3.31	3.65	0.64	1.01	1.04	0.10	yes
	T_C_	95.91	3.72	3.88	0.68	97.75	2.98	3.05	0.54	1.56	1.02	0.10	yes
	DHAA	8.48	1.28	15.14	1.85	7.08	3.71	52.35	6.21	8.35	0.84	1.01	yes
BCR431	AA	398.60	12.02	3.02	0.66	390.14	6.14	1.57	0.34	3.83	0.98	0.07	yes
	T_C_	478.48	14.88	3.11	0.70	490.52	10.45	2.13	0.48	2.03	1.03	0.08	yes
	DHAA	79.87	16.96	21.23	3.63	100.38	11.62	11.58	2.05	2.13	1.26	0.46	yes

* s—standard deviation. H—Horrat parameter. F—parameter of Snedecor’s F-test, P—the ratio of means of determination results. U—uncertainty for *p* value.

**Table 2:** Analysis of the accuracy of the analytical method based on the determination of the certified reference material BCR 431.

BCR 431							
	
X_CRM_ *	u_CRM_ *		X_m_ *	u_m_ *	Δm *	U_Δ_ *	Accuracy
(mg/100 g)	(mg/100 g)		(mg/100 g)	(mg/100 g)	(mg/100 g)	(mg/100 g)	
483	9.8	HPLC-TCEP	478	6.1	4.5	23.1	yes
		HPLC-THP	491	4.3	7.5	21.4	yes

* X_CRM_—the certified value, u_CRM_—uncertainty of the certified value, X_m_—the mean value measured, u_m_—uncertainty of the result of a measurement (expressed as the standard deviation of measurement series), Δm—the absolute difference between the mean measured value and the certified value, U_Δ_—expanded uncertainty of difference between the measured value and the certified value.

## Data Availability

Not applicable.

## Sample Availability

Samples of the compounds are not available from the authors.
